# Acute Kidney Injury: Advances in Clinical Management

**DOI:** 10.3390/jcm11247308

**Published:** 2022-12-09

**Authors:** Antonio Lacquaniti, Paolo Monardo

**Affiliations:** Nephrology and Dialysis Unit, Department of Internal Medicine, Papardo Hospital, 98158 Messina, Italy

Acute kidney injury (AKI), closely related to increased mortality, involved 15–20% of hospitalized patients with higher incidence, with about 50% in the intensive care unit (ICU) [1,2]. Starting from these serious data, almost one decade ago, the International Society of Nephrology, recently echoed by the European Renal Association, launched the “0 by 25” project to eliminate preventable deaths from AKI by 2025 in low- and high-income countries [3,4]. However, after almost ten years from the start of this challenging project, the question is: are we prepared to reach this result in the remaining five years? The awareness problem among clinicians and knowledge about physiopathological mechanisms and pathways activated in AKI must be improved, notwithstanding the progress made in recent years in biotechnology and therapies.

The precocious diagnosis is crucial in clinical practice, such as the distinction between transient and persistent AKI, with obvious consequences for the patient during hospitalization [5]. The first step is to identify the renal disease, and be aware of the definition of AKI, described as an abnormality in kidney structure or function that has existed for fewer than 90 days, at which point chronic kidney disease (CKD) begins [6,7]. This condition reflects not only kidney damage, but also a systemic involvement of different organs and diseases (heart failure, liver failure, and sepsis), which themselves cause substantial morbidity and mortality [8].

Its pathophysiology varies according to the myriad of conditions associated with its development, and further studies are needed to analyze the mechanisms of AKI and to develop personalized and targeted therapies. Cardiac surgery with cardiac bypass is the classic example of AKI, with pathophysiology based on various interrelated mechanisms, starting from inflammatory and ischemic processes leading to tubular cell injury [9]. Conversely, low cardiac output and renal vein congestion characterized the cardio-renal syndrome type I (acute coronary syndrome leading to heart and kidney acute failure), affecting kidney perfusion pressure, while compensatory mechanisms can become insufficient to maintain blood flow autoregulation. At the same time, sepsis is the most common trigger of severe AKI in ICU patients, and vascular dysfunction may play a vital role in the pathophysiological mechanisms [10]. However, in animal models of septic AKI, the renal blood flow increases above normal levels, and renal histopathology in the first 48 h is indistinguishable from the healthy group, with a decreased glomerular filtration rate (GFR), based on efferent arteriolar vasodilatation and intrarenal shunting, contributing to a decreased medullary oxygenation [11]. These findings support the need to measure renal blood flow in septic patients through, for example, the renal Doppler ultrasound, which can non-invasively explore kidney hemodynamics, measuring the renal resistive index (RI), reflecting the alterations in the blood flow profile of the intrarenal arteries. However, to date, there is a debate if RI might help to distinguish and predict transient from persistent AKI compared to usual clinical variables, such as serum creatinine and the non-renal Sequential Organ Failure Assessment (SOFA) Score [12,13,14].

Nevertheless, if the mechanism of renal damage depends on the primary cause, early recognition of AKI is the starting point to optimize the management and prevent deterioration of kidney function, and the assessment of individual clinical risk needs wider dissemination among clinicians to allow for the identification of patients at high risk.

AKI is a clinical diagnosis, and it is unlikely that any single kidney injury marker will fully predict the changes in kidney function in this complex condition. Although various biomarkers have been associated with AKI and adverse outcomes, the clinical application of any single biomarker has failed to demonstrate troponin-like diagnostic performance in myocardial infarction. Recently, a clinical immunoassay for the detection of insulin-like growth factor binding protein 7 (IGFBP7) and tissue inhibitor of metalloproteinases-2 (TIMP-2), the NephroCheck test (Astute Medical), received US Food and Drug Administration approval for AKI risk assessment in critically ill patients, showing sensitive, specific, and highly predictive properties in AKI adults and children after cardiac surgery [15]. These biomarkers have been associated with long-term outcomes after AKI, predicting renal recovery or the dialysis start [16,17].

While cardiologists have electrocardiogram alterations and troponin levels to define acute heart damage accurately and precociously, nephrologists base all clinical activities and decisions on the changes in serum creatinine (sCr) or urinary output, which are neither sensitive nor specific for AKI. Several sCr-based classification systems define AKI, but changes in sCr lack sensitivity because nearly 50% of GFR must be lost before sCr increases.

How could we diagnose AKI precociously if we have no biomarkers detecting this subclinical stage of AKI? The latter represents the renal functional reserve (RFR), in which the adaptive nephron hypertrophy replies to an acute reduction in the number of functioning nephrons. The capacity of this RFR mechanism diminishes as renal functional mass declines, leading to a gradual rise in SCr, but at least after 48–72 h since the damage started [18]. If creatinine does not provide sufficient guarantees as a reliable marker, changes in urine output might be more sensitive, but appear less specific, as observed during polyuria, due to defects in tubular urine concentration ability in acute interstitial nephritis. Starting from these assumptions, the 23rd ADQI consensus meeting proposed combining clinical assessments, traditional tests, and validated novel biomarkers to identify patients at risk of AKI [19].

Considering the complex and multifactorial causes, a panel of multiple biomarkers of renal stress, injury, and kidney reserve function could provide better discrimination for AKI. Furthermore, more kidney tissue-specific markers may help localize and quantify the severity of AKI, providing a deeper understanding of the pathophysiology of AKI, offering opportunities for personalized management of AKI, and supporting a re-analysis of the existing AKI criteria [20].

In the last decade, the computer decision support improved the diagnostic approach to AKI and its treatment, acting on the awareness problem among clinicians. This system might improve outcomes, reduce nephrotoxic medication and intravascular radiocontrast exposure, increase consultations for nephrology and critical care medicine [2].

If the diagnostic approach to AKI is complex and debated, notwithstanding advances in biotechnologies, the pharmacotherapy for AKI needs to be improved, avoiding schematic protocols, and targeting a personalized strategy. Aki leads to electrolyte and acid-base disorders, fluid accumulation, and metabolic dysfunction, impairing the immune system and reducing the patient’s ability to clear infection [21,22]. Metabolic acidosis is commonly observed in AKI patients, representing an indication of bicarbonate infusion with its beneficial effects in severe metabolic acidosis in association with AKI, as revealed by a randomized controlled trial involving more than three hundred ICU patients [23]. Furthermore, regardless of metabolic acidosis, several data analyzed the effects of the administration of prophylactic isotonic sodium bicarbonate infusion, to prevent contrast-induced nephropathy (CIN), with the potential reduction of renal oxygen consumption and free oxygen radicals mediated by bicarbonate. However, multicenter randomized controlled trials have shown that N-acetylcysteine and bicarbonate do not provide additional protection beyond hydration therapy in contrast-associated AKI [24,25].

Biomarker-driven application of specific bundles derived from KDIGO recommendations or structured organization of nephrologist teams has permitted reductions in the occurrence of severe AKI cases, the requirement of renal replacement therapy (RRT), and the length of ICU stay, acting on fluid management, titrating vasoactive medication and maintenance of perfusion pressure [26,27]. In some patients, AKI is severe enough to require RRT, but no univocal criteria exist to justify such interventions, the best time to start the RRT, and the type of dialytic prescription (intermittent or continuous therapy). However, clinicians must consider factors like hemodynamic conditions, cardiorespiratory instability, potassium levels, fluid status, acid-base status, creatinine and urea levels, urine output, and other complications [28].

The approach in these patients should be based on qualitative strategies rather than a “quantitative belief” underlying that “the more, the better”, referring to the high dose of RRT and the highly intensive dialytic treatments, did not improve clinical outcomes [29,30].

Defining the intent and goals of kidney support therapy is the principal consideration when deciding to start the RRT with classical parameters, such as metabolic acidosis, hyperkalemia, or severe fluid overload, which could only marginally depict a complex patient with AKI. A ”modern” indication of RRT is to remove pro-inflammatory cytokines, pathogen and damage-associated molecular patterns, pathogen agents, and endotoxin molecules behind traditional parameters, taking advantage of new hemofilters with enhanced biotechnologies in membrane and adsorbent structures. Only personalized therapy could avoid negative results from this approach, identifying specific patient subgroups.

In conclusion, if the clinical management starts from the identify patients at risk of AKI, analyzing concomitant and triggering diseases, the attention of the nephrology must not finish after the resolution of this complex condition, neglecting patients at high risk of CKD. A protracted enhancement of function in remnant nephrons during AKI represents a maladjustment in a long period, with a progressive and chronic injury leading to glomerular sclerosis, hyalinosis, and fibrosis with progressing CKD [31]. Unfortunately, most patients do not receive nephrology follow-up after AKI, suggesting the opportunity to improve care by close follow-up in designated outpatient clinics, where nephrologists may stratify and reduce the risk for CKD progression, acting through specific medical interventions [32].
